# Supplementary material for: Measuring the work environment among healthcare professionals: Validation of the Dutch version of the Culture of Care Barometer
Source: PLoS One. 2024 Feb 29;19(2):e0298391. doi: 10.1371/journal.pone.0298391 (PMC10903908; doi:10.1371/journal.pone.0298391)
Supplement: S1 Table — (PDF) [file pone.0298391.s001.pdf]

Sample characteristics of content validity on comprehensibility

Profession	n	%
Physicians	4	11%
Nurses	10	28%
Management	7	19%
Support staff	3	8%
Research	3	8%
Policy advisors Q&S	9	25%
Total	36	100%
