# Supplementary material for: Measuring the work environment among healthcare professionals: Validation of the Dutch version of the Culture of Care Barometer
Source: PLoS One. 2024 Feb 29;19(2):e0298391. doi: 10.1371/journal.pone.0298391 (PMC10903908; doi:10.1371/journal.pone.0298391)
Supplement: Attachment — Submitted filename: Comment Reviewer Measuring the work environment among healthcare professionals.pdf [file pone.0298391.s005.pdf]

Measuring the work environment among healthcare professionals: Validation of the Dutch version of the Culture of Care Barometer

Comments to the Author

Congratulations on the submitted manuscript. The topic is timely and will be of interest to the readers of the journal. However, a few changes are suggested to improve the clarity of this manuscript. I have several recommendations and questions about the manuscript.

No	Page	Descriptions of item	Comment
		**Title**<br>Measuring the work environment among healthcare professionals: validation of the Dutch version of the Culture of Care Barometer	Capital Validation(V)
1.		**Abstract**<br><br>WE<br><br>**Keyword**	What are the inclusion and exclusion criteria?<br><br>Please state the full meaning. For example, in the first paragraph.<br>Example: A positive work environment (WE) is paramount for healthcare employees to provide good quality care.<br><br>Too many keywords (10). Please identify the important keyword for this study.
2	10	**Introduction**	Need to explain first what Health literacy is.<br><br>Correct this. (Sajjadi et al)
3.	16	**Results**<br><br>0,30	For the tables, write the symbol.<br>0.30
4.		**Discussion**<br><br>Three hypotheses were rejected because the CoCB-NL performed better than expected. Contrary to the expected moderate correlations, strong	Recommendation:<br>Make the discussion based on subheadings clearer to the reader.<br><br>Please harmonize this statement.

		correlations were found between factors of the CoCB-NL and the SAQ-NL	
5.		**Recommendation**	Recommend adding the subheading for recommendations for this study.
6.		References	Most of the citations are above 5 years. Very good.
