# Supplementary material for: Measuring the work environment among healthcare professionals: Validation of the Dutch version of the Culture of Care Barometer
Source: PLoS One. 2024 Feb 29;19(2):e0298391. doi: 10.1371/journal.pone.0298391 (PMC10903908; doi:10.1371/journal.pone.0298391)
Supplement: Attachment — Submitted filename: Response to Reviewers.docx [file pone.0298391.s004.docx]

*Rebuttal letter*

Dear Prof. Ulutaş Deniz

We are pleased to learn that PLOS ONE is interested in publishing our paper ‘*Measuring the work environment among health care professionals: validation of the Dutch version of the Culture of Care Barometer’*. We thank both reviewers for providing us with the positive feedback and constructive suggestions to improve our manuscript. We have revised our manuscript accordingly. Below we provide a point-by-point reaction to the comments of each reviewer.

Journal Requirements

*1. Please ensure that your manuscript meets PLOS ONE's style requirements, including those for file naming.*

We have reviewed the manuscript for style compliance and adjusted the paper to adhere to the style requirements of PLOS ONE. We adjusted the supporting information from one file into three separate files with corresponding file names.

*2. We note that you have stated that you will provide repository information for your data at acceptance. Should your manuscript be accepted for publication, we will hold it until you provide the relevant accession numbers or DOIs necessary to access your data. If you wish to make changes to your Data Availability statement, please describe these changes in your cover letter and we will update your Data Availability statement to reflect the information you provide.*

In our Data Availability Statement we have selected the box ‘*No - some restrictions will apply’*.
All data underlying reported findings are anonymized deposited with the Tilburg University data repository and access can be granted by either the authors or the data steward of Tilburg University ([datasteward@tilburguniversity.edu](mailto:datasteward@tilburguniversity.edu)), who will ask for permission of the Erasmus MC Board of Directors as they are the owners of the data. The accession number is currently pending, but we will of course provide it as soon as it is known. We expect to have that settled this month (September 2023).

*3. Please review your reference list to ensure that it is complete and correct. If you have cited papers that have been retracted, please include the rationale for doing so in the manuscript text, or remove these references and replace them with relevant current references. Any changes to the reference list should be mentioned in the rebuttal letter that accompanies your revised manuscript. If you need to cite a retracted article, indicate the article’s retracted status in the References list and also include a citation and full reference for the retraction notice.*

Thank you for your check on the reference list. We have replaced the retracted reference (*No. 37: Huebner LA, Zacher H. Effects of Action Planning After Employee Surveys. J Pers Psychol. 2022;21(1):23-36.)* with the correct reference from the same authors (*Huebner LA, Zacher H. The role of mean item ratings, topic distance, direct leadership, and voice climate in action planning after employee surveys. Acta Psychol (Amst). 2023;238:103950. PubMed PMID: 37379784*).
In addition, the reference of Guo et al. (2022; #27) has been updated from the 'EPub reference’ to the final reference of the paper.
Both changes are not visible with track changes in the revised version because the whole reference list has been re-uploaded via Endnote.

Editor Comments:

*Manuscript ID PONE-D-23-07783 entitled "Measuring the work environment among healthcare professionals: validation of the Dutch version of the Culture of Care Barometer" which you submitted to the PLOS ONE, has been reviewed. The comments of the reviewer(s) are included at the bottom of this letter.*

*The reviewer(s) have recommended publication, but also suggest some revisions to your manuscript. Therefore, I invite you to respond to the reviewer(s)' comments and revise your manuscript.*

We thank the editor, Prof. Ulutaş Deniz, for the positive reaction. We will respond to the reviewers suggestions below and revise our manuscript as suggested by the reviewers.

Reviewer 1:

*Recognising the centrality of a work environment that cares for and values its workforce for recruiting and retaining the workforce, achieving a high-performing organisation, and for safe and high-quality patient care, the aim of this study was to test the psychometric properties of a Dutch version of the Culture of Care Barometer.*

We thank reviewer 1 for this critical reflection on our objective. Indeed, this is exactly what we aim to do.

*The abstract was presented using headers and core details were included. There was no reference to a methodological framework for conducting this study.*

We have used a structured form to present our abstract, we thank reviewer 1 for pointing this out.

Indeed, we do not refer in the abstract to a methodological framework for conducting this study. We used the COnsensus-based Standards for the selection of health Measurement Instruments (COSMIN) framework for measurement properties in this study and applied both the COSMIN study design checklist [1]{Mokkink, 2019 #34} and the reporting guideline [2]. PLOSONE’s reporting guidelines prescribe that references should not be included in the abstract. Therefore, we did not include the abbreviation COSMIN in the abstract. However, we do describe in the abstract the measurement properties that we have investigated with this study (content validity, structural validity, internal consistency, hypothesis testing for construct validity, and responsiveness). In the paper the COMSIN methodology and explanation of the various measurement properties are presented and explained in line 95-96, Figure 1 and lines 130-131, 141-142, 195-212. We trust that this explanation will be sufficient for the reviewer.

*The Introduction was succinct in contextualising this study- what characterises a positive work environment and why a positive work environment has positive outcomes for staff, patients, and wok organisations.*

We thank reviewer 1 for this compliment.

*As a foundation for this study the authors conducted a systematic review on the psychometric evaluation of instruments measuring the work environment of healthcare professionals in hospitals, this identified the Culture of Care Barometer (Rafferty et al 2017) as an instrument that was worthy of further investigation to determine its validity and reliability for use in the Netherlands. Following on from this was a Delphi study by the authors that culminated in confirmation of 36 items by the experts.*

We thank reviewer 1 for this reflection on our approach.

*For cross-cultural validation, the content and structural validity and internal consistency of the CoCB-NL were examined. The instrument was further validated by assessing convergent validity, discriminant validity, and responsiveness.*

*The design involved translation and cultural adaptation and a longitudinal survey to confirm structural validity and internal consistent, testing hypotheses to determine the convergent and discriminant validity and assessment of responsiveness.*
 *The study was developed and reported according to the Consensus-Based Standards for the Selection of Health Measurement Instruments (COSMIN) study design checklist [19] and reporting guideline [22].*

We thank reviewer 1 for the reflection on our methodology used for the validation of the CoCB-NL.

*2 Dutch hospitals participated- the sampling approach was not reported- and a convenience sample of seven departments were recruited.*

We acknowledge that the sampling approach for the two participating Dutch hospitals is not reported. We used the professional network of our research team members and purposively approached the hospitals that participated in our study as these hospitals were planning to improve their work environment. We have added this information in our manuscript (line 133-134).

*The process for examining the content validity of the instrument was presented clearly and in full.*

We are grateful for these compliments.

*The questionnaire survey, comprising the CoCB-NL, Work engagement scale, and Safety attitudes questionnaire were distributed electronically to a total population sample of 1730 employees, achieving a response rate of 63.2%, with 1062 complete cases. Survey 2 was completed by 590 respondents (48.4%).*

We thank reviewer 1 for the reflection on our survey.

*Results for the different components of psychometric testing were presented clearly using Tables and narrative. The authors concluded that the CoCB-NL is sufficiently valid and reliable for assessing employees’ perceptions of their work environment and there was goodness of fit for a four- and five- factor model. Taking this forward to examine and improve workplace culture is imperative for the reasons outlined above and addressed in the manuscript.*

We appreciate the acknowledgment of our efforts in clearly presenting the results of the psychometric testing.

Reviewer 2:

*The authors provide a comprehensive demonstration of the validity and reliability of the CoCB-NL as an effective tool for assessing employers' perceptions of their work environment.*

We thank reviewer 2 for the insightful feedback on our study. We are gratified that the reviewer recognizes the comprehensive nature of our demonstration of the validity and reliability of the CoCB-NL as a valuable instrument for evaluating employees' perceptions of their work environment.

*While the methods employed are generally well-presented, further information is needed in order to replicate this study successfully. Specifically, the authors should clarify the sampling method used and enhance transparency regarding the research team responsible for content validation and its corresponding results.*

We acknowledge the reviewer's concern regarding the replicability of our study and the necessity for additional information. Hence, we provided more detailed information of our sampling approach. The sample for translation and the content validation is clarified more clearly in line 142, 149-150. The supporting information ‘S1 Table’ includes descriptive data on the content validity sample. We have now referred to this more explicitly in line 152.

Furthermore, we understand the reviewer's call for increased transparency regarding the research team responsible for content validation and the corresponding results. Initially, we included the table with this information in a supplementary file. To enable readers to gain a more comprehensive understanding of the study's execution and results, we have replaced this table into the manuscript (line 161, table 1).

Subsequently, we have changed the numbering of the tables.

We greatly appreciate the reviewer's valuable input, which helps us refine the presentation and accessibility of our research.

We hope these revisions will make our manuscript suitable for publication in your journal.

Sincerely, on behalf of all co-authors,

1.	Mokkink LB, Prinsen CAC, Patrick DL, Alonso J, Bouter LM, de Vet HCW, et al. COSMIN Study Design checklist for Patient-reported outcome measurement instruments2019 08-25-2023:[1-32 pp.]. Available from: [https://www.cosmin.nl/wp-content/uploads/COSMIN-study-designing-checklist_final.pdf#](https://www.cosmin.nl/wp-content/uploads/COSMIN-study-designing-checklist_final.pdf).

2.	Gagnier JJ, Lai J, Mokkink LB, Terwee CB. COSMIN reporting guideline for studies on measurement properties of patient-reported outcome measures. Qual Life Res. 2021;30(8):2197-218. PubMed PMID: 33818733.
