# Supplementary material for: Measuring the work environment among healthcare professionals: Validation of the Dutch version of the Culture of Care Barometer
Source: PLoS One. 2024 Feb 29;19(2):e0298391. doi: 10.1371/journal.pone.0298391 (PMC10903908; doi:10.1371/journal.pone.0298391)
Supplement: Attachment — Submitted filename: Response to Reviewers.docx [file pone.0298391.s006.docx]

Rebuttal letter	January 11, 2024

Dear, Professor Ulutaş Deniz

We are pleased to learn that the revisions to our paper ‘Measuring the work environment among health care professionals: validation of the Dutch version of the Culture of Care Barometer’ has been well received by you and both reviewers. We thank you and both reviewers for providing us with previous and current constructive feedback which helped us to improve our paper. We have revised our manuscript accordingly. Below we provide a point-by-point reaction to the comments.

Journal Requirements:

*Please review your reference list to ensure that it is complete and correct. If you have cited papers that have been retracted, please include the rationale for doing so in the manuscript text, or remove these references and replace them with relevant current references. Any changes to the reference list should be mentioned in the rebuttal letter that accompanies your revised manuscript. If you need to cite a retracted article, indicate the article’s retracted status in the References list and also include a citation and full reference for the retraction notice.*

We have reviewed the reference list, no alterations are needed anymore. In the previous revision we replaced the retracted article with another relevant paper. To address feedback below we have added a reference to the reference list: Brubakk et al., 2021. Ref no: 37.

Editor Comments:

*Manuscript PONE-D-23-07783R1 titled " Measuring the work environment among healthcare professionals: validation of the Dutch version of the Culture of Care Barometer’ which you submitted to PLOS ONE, has been reviewed. The comments of the reviewer(s) are included at the bottom of this letter.*

*The reviewers suggest some minor revisions to your manuscript. Therefore, I invite you to respond to the reviewer(s)' comments and revise your manuscript.*

We thank the editor for the review and the invitation to address the minor revisions as suggested by the reviewers. We hope that our response is satisfactory.

Reviewer 2

We thank you for the constructive feedback and are pleased that the revisions that we have made meet the expectations.

*Addressing my concerns and clarifying them strengthens the value of disseminating the findings. Consider knowledge translation programs for a broader impact.*

Commensurate the reviewer we believe that the Culture of Care Barometer has much potential for practical application in the daily practice of healthcare organizations. We therefore already have planned knowledge translation activities to disseminate the findings and bridge the gap between research and practice, e.g. final report summaries, presentations at conferences, workshops, a web page and press releases.

Reviewer 3

We thank you for the positive and constructive feedback. We have addressed the points raised by the reviewer.

*Title
Measuring the work environment among healthcare professionals: validation of the Dutch version of the Culture of Care Barometer Capital Validation(V)*

Thank you for noticing. We have changed this.

*Abstract:
WE: Please state the full meaning. For example, in the first paragraph. Example: A positive work environment (WE) is paramount for healthcare employees to provide good quality care.*

*What are the inclusion and exclusion criteria?*

Thank you for noticing. We have added the abbreviation between brackets and information on the inclusion criteria for participating departments.

*Keyword
Too many keywords (10). Please identify the important keyword for this study.*

We have identified five most reflective keywords for our study: ‘working conditions’, ‘organizational culture’, ‘hospitals’, ‘validation study’ and ‘questionnaire’.

*Introduction
Need to explain first what Health literacy is.
Correct this. ( Sajjadi et al)*

We have not used the concept ‘health literacy’ nor the reference to Sajjadi et al in the introduction or study. Please, can the reviewer explain what is meant by this suggestion?

*Results
0,30: For the tables, write the symbol. 0.30*

Thank you for noticing. We have exchanged the comma’s for dots in table 1 and checked the other tables for inconsistencies.

*Discussion
Recommendation: Make the discussion based on subheadings clearer to the reader.*

Thank you for your suggestion, we now have added subheadings to enhance the readability.

*Discussion:
Three hypotheses were rejected because the CoCB-NL performed better than expected. Contrary to the expected moderate correlations, strong correlations were found between factors of the CoCB-NL and the SAQ-NL. Please harmonize this statement.*

We agree with the reviewer that our formulation was not correct. We rephrased the statement into: ‘*Three hypotheses were rejected because contrary to the expected moderate correlations, strong correlations were found between factors of the CoCB-NL and the SAQ-NL. This is commensurate findings of Brubakk et al. [37] who found that positive work environments associate with a positive safety climate and favorable patient safety outcomes.’(lines 334-338).*

*Recommend adding the subheading for recommendations for this study.*

We have now added ‘recommendations’ to the conclusion subheading.

*References
Most of the citations are above 5 years. Very good.*

Thank you for this remark.

*While revising your submission, please upload your figure files to the Preflight Analysis and Conversion Engine (PACE) digital diagnostic tool,* [*https://pacev2.apexcovantage.com/*](https://eur01.safelinks.protection.outlook.com/?url=https%3A%2F%2Fpacev2.apexcovantage.com%2F&data=05%7C02%7Cs.maassen%40erasmusmc.nl%7Ce7afa46bcd024ef4f3c008dc0126e9bd%7C526638ba6af34b0fa532a1a511f4ac80%7C0%7C0%7C638386513995581164%7CUnknown%7CTWFpbGZsb3d8eyJWIjoiMC4wLjAwMDAiLCJQIjoiV2luMzIiLCJBTiI6Ik1haWwiLCJXVCI6Mn0%3D%7C3000%7C%7C%7C&sdata=TP0w53fgpvUCnb4%2BuF6QNNJIBjomtI4f5Qlgj3nXv6w%3D&reserved=0)*. PACE helps ensure that figures meet PLOS requirements. To use PACE, you must first register as a user. Registration is free. Then, login and navigate to the UPLOAD tab, where you will find detailed instructions on how to use the tool. If you encounter any issues or have any questions when using PACE, please email PLOS at* [*figures@plos.org*](mailto:figures@plos.org)*. Please note that Supporting Information files do not need this step.*

We have checked the figures with PACE.

We greatly appreciate the reviewer's valuable input, which helped us refine the presentation and accessibility of our research.

We hope these revisions will make our paper suitable for publication in your journal.

Sincerely, on behalf of all co-authors,

Susanne Maassen
